# Benchmarks and methods for 3D medical image retrieval

**DOI:** 10.1038/s41598-026-38473-z

**Published:** 2026-04-06

**Authors:** Asma Ben Abacha, Alberto Santamaría-Pang, Ho Hin Lee, Jameson Merkow, Qin Cai, Surya Teja Devarakonda, Abdullah Islam, Julia Gong, Matthew Lungren, Reza Forghani, Ankush Jindal, Thomas Lin, Noel C. F. Codella, Ivan Tarapov

**Affiliations:** 1 Health and Life Sciences AI, Microsoft, Redmond, WA USA; 2 AdventHealth Medical Group, Maitland, FL USA; 3 Johns Hopkins School of Medicine, Baltimore, MD USA

**Keywords:** 3D Image Search, Data Creation, Multi-modal Models, Radiology, Image processing, Data publication and archiving, Computational models, Machine learning

## Abstract

The increasing use of medical imaging in healthcare settings presents a significant challenge due to the additional workload for radiologists, yet it also offers opportunity for enhancing healthcare outcomes if effectively leveraged. Artificial Intelligence (AI)-based 3D medical image retrieval holds the potential to alleviate radiologists’ burden by offering evidence-based diagnostics and predictions that can enhance the scale and accuracy of radiologists’ work, while simultaneously supporting output verification for safety and regulatory compliance. Despite its promise, the field of 3D medical image retrieval lacks established evaluation benchmarks, comprehensive datasets, and rigorous evaluation studies. This paper aims to address these gaps by introducing the first benchmark for 3D Medical Image Retrieval (3D-MIR) and evaluating various pre-trained models and implementation approaches for retrieval. The benchmark includes four anatomies (Liver, Colon, Pancreas, and Lung) imaged using computed tomography (CT). A range of 3D image search strategies are explored, including those that use aggregated 2D slices/3D volumes (Image-to-Image) and text embeddings from popular foundation models as queries (Text-to-Image). Additionally, novel multi-modal and supervised fine-tuning approaches are investigated to generate multi-modal embeddings for 3D image retrieval. The paper provides quantitative and qualitative assessments of each approach, along with an in-depth discussion offering insights for future research and solutions to support clinical decision-making and healthcare applications. To foster advancement in this field, our benchmark, models, and code are made publicly available.

## Introduction

Medical imaging workloads have been increasing faster than the supply of specialists, contributing to increased radiologist burnout^1^. AI has the potential to be leveraged to scale and refine clinical decision-making, thereby improving clinical productivity and mitigating these challenges, primarily through harnessing extensive databases of imaging data and clinical histories. The key question is: *How can imaging databases best be leveraged as a resource to build AI systems to enhance clinician productivity?*

**Fig. 1:**
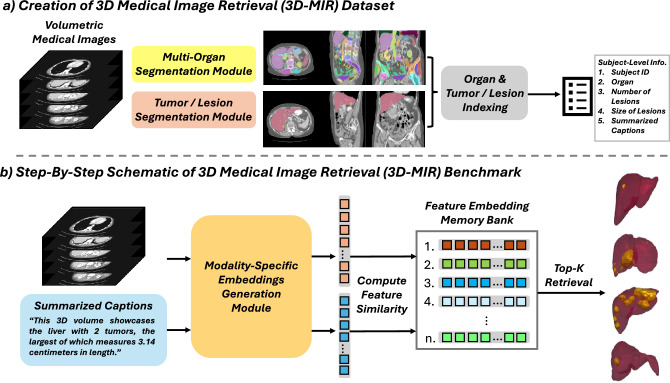
Workflow for creating the 3D medical image retrieval (3D-MIR) dataset and benchmark. (a) The creation of the 3D-MIR dataset starts with volumetric medical images, which are segmented using both a multi-organ segmentation module and a tumor/lesion segmentation module. The segmentation outputs provide localized information about organs and lesions, which is then used to index subject-level details, such as organ type, lesion count, lesion size, and summarized captions. (b) The schematic for the 3D-MIR benchmark illustrates how summarized captions and volumetric images are processed through a modality-specific embeddings generation module. These embeddings are stored in a feature embedding database. During retrieval, feature similarity is computed, allowing for ranked order retrieval, thereby enabling efficient and contextually aware multi-modal image search.

One class of approaches that are being explored in this regard is built for retrieving relevant and specific information from imaging databases. The motivation for this is two-fold: 1) data retrieval can be used to yield evidence-based diagnostics and predictions, supporting improvements in scale and providing accurate diagnostics, while simultaneously enabling clinician verification for safety and regulatory purposes, 2) data retrieval can support education and research to advance scientific efforts and improve healthcare services. Figure 1 illustrates an example of 3D Medical Image Retrieval (3D-MIR), specifically within CT volumes. First, a database is constructed from imaging volumes, data analysis techniques (such as organ segmentation), and associated metadata. Uni-modal or multi-modal queries can then be indexed into this database, whereby the system then presents ranked search results.

**Fig. 2:**
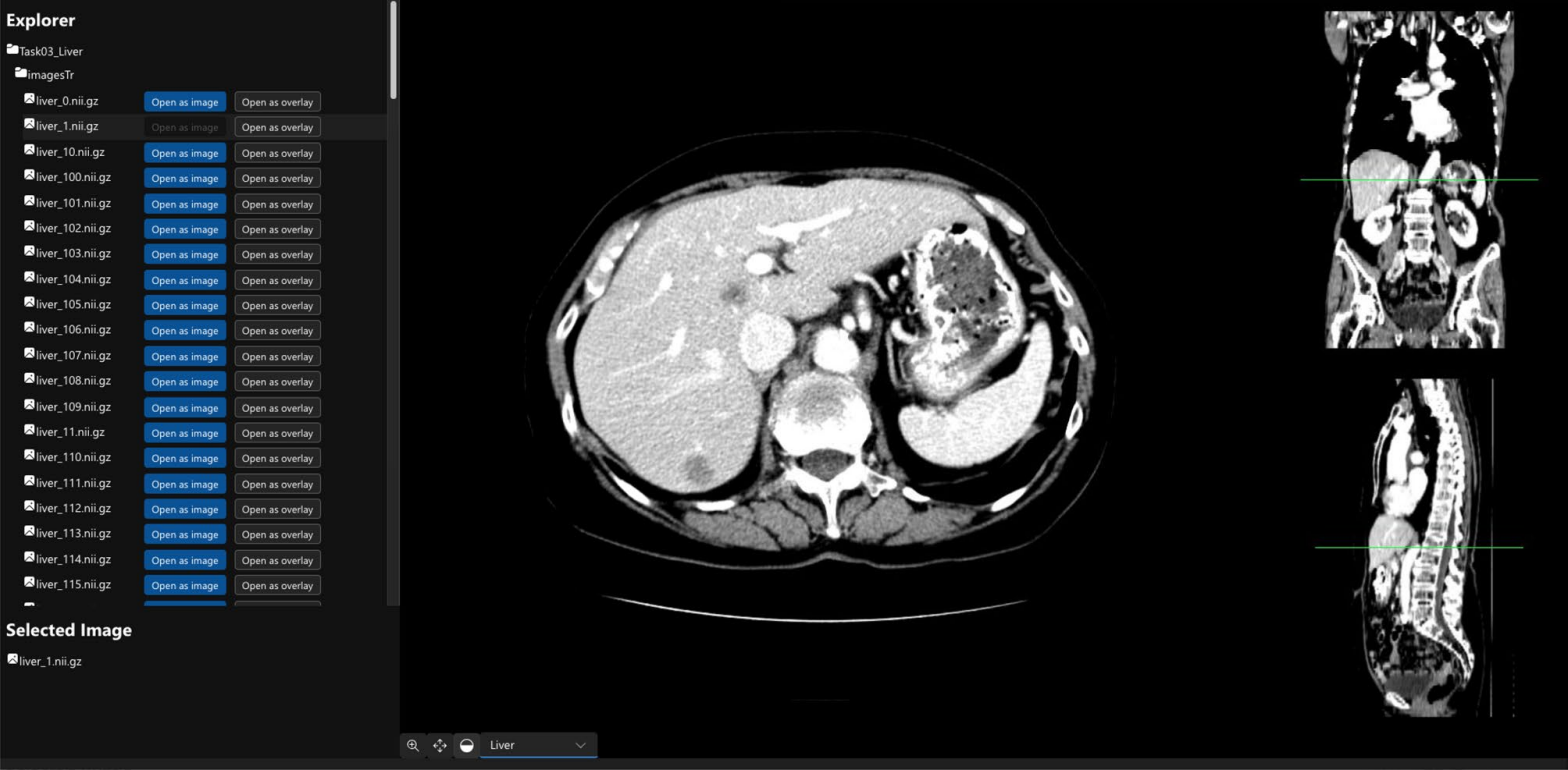
Annotation interface used to validate the tumor flag and group annotations.

Despite the significant potential of 3D medical image retrieval, the field remains underexplored, largely due to the absence of standardized benchmarks and evaluation protocols. To address this gap, we present the first 3D Medical Image Retrieval (3D-MIR) benchmark, spanning four distinct organ systems imaged via computed tomography (CT). Alongside this benchmark, we introduce novel multi-modal and supervised fine-tuning approaches for generating joint image–text embeddings specifically optimized for 3D retrieval. We conduct a comprehensive comparative study of multiple retrieval strategies, leveraging state-of-the-art medical imaging foundation models, and provide both quantitative metrics and qualitative assessments. These results yield key insights into the strengths and limitations of each approach and help establish a foundation for future research. To support continued progress in this domain, we publicly release our benchmark dataset, annotation tools, and fine-tuned models.

Our work lays the groundwork for intelligent systems that enable radiologists to efficiently search and retrieve clinically relevant 3D scans, turning complex imaging data into actionable decision-support resources. This advancement has the potential to streamline diagnostic workflows and catalyze further innovation in intelligent data management across healthcare.

**Our key contributions are as follows:**

- We introduce a multi-region dataset of 3D CT scans, enabling consistent evaluation across multiple anatomical categories.
- We release a complete benchmark, including the dataset, evaluation code, and baseline models to promote reproducibility and further research.
- We propose novel multi-modal and supervised fine-tuning approaches to generate multi-modal embeddings for 3D image retrieval.
- We perform a systematic empirical study comparing a range of retrieval strategies, from 2D slice aggregation to 3D volumetric encoders and large-scale vision-language models.

The paper is organized as follows: In Section “Related work”, we review related work in medical image retrieval and multi-modal representation learning. Section "3D-MIR dataset creation" introduces the 3D-MIR benchmark, including dataset construction and labeling strategy. In Section “Retrieval methods”, we present our retrieval methods, including Image-to-Image search methods, Text-to-Image methods, and our proposed Multi-modal Search Methods (M2ST) framework. Section “Experiments” details the experimental setup and provides a comprehensive analysis of retrieval performance across different anatomical regions. We present an expert-based clinical relevance evaluation in Section "Clinical relevance evaluation", discuss key findings and limitations in Section “Discussion”, and conclude the paper in Section “Conclusion”.

## Related work

3D medical imaging has predominantly concentrated on tasks such as classification^2–6^, detection^6–8^, and segmentation^9–14^, while issues pertaining to diagnosis based retrieval remain under-explored. Simonyan *et al.*^15^ reported the first method for 3D Region of Interest (ROI) retrieval in medical imaging. Results are demonstrated in ROI Atrophy-Aware Brain MRI Retrieval, where an atlas-based model is used to compare vulnerable brain areas (e.g., hippocampal deterioration). For a comprehensive review of image retrieval systems using deep learning and hand-crafted features in medical imaging see Vishraj *et al.*^16^.

Although extensive research has been conducted on text-based image retrieval, highlighted in various studies^17–21^, and medical image captioning^22–25^, the integration of these techniques with the latest large-scale foundation models^26–29^ presents a significant opportunity for advancement. However, their application in 3D multi-modal medical image retrieval is still in its infancy^30^. Current public benchmarks in 3D medical imaging, primarily focused on segmentation (e.g., MSD^11^, BTCV^9^, DenseVNet-MultiOrgan^10^, TCIA Pancreas-CT^31^, KiTS19^32^, RAD-ChestCT^33^, ATLAS v2.0^34^, EPISURG^35^) and classification (e.g., MedFMC^2^, MedMNIST^3^, BenchMD^4^), do not fully address the needs of 3D image retrieval.

To address this gap, we introduce the first public benchmark, 3D-MIR, tailored for 3D multi-modal information retrieval. This benchmark includes essential elements such as full volume data, 3D labels, localized metrics at the organ level, and textual descriptions of organ lesions. Recognizing the time-intensive nature of 3D labeling and the generation of medical reports, we leverage public datasets such as the Medical Segmentation Decathlon (MSD)^11^ for lesion segmentation, along with open-source organ segmentation models like TotalSegmentator^12^ built on nnU-Net^36^ to localize lesions in organs. We then quantify 3D lesion morphology at the organ level and utilize OpenAI’s GPT foundation model^37^ for describing organ characteristics, such as the number of lesions in the liver. Our source code, data generation methods, and benchmark details are made publicly available: https://github.com/abachaa/3D-MIR.

## 3D-MIR dataset creation

In this section, we elaborate on the development of the 3D-MIR multi-organ benchmark. Our aim is to harness public datasets that feature 3D lesion annotations to provide detailed morphological descriptions of these lesions in the context of their associated organs. These descriptions are then used to facilitate measuring relevance between query and retrieved results in retrieval settings.

### Source data

The 3D-MIR dataset is constructed using the Medical Segmentation Decathlon (MSD) dataset^11^, which offers 10 publicly available cohorts. Our research particularly focuses on the chest-CT cohort from the MSD, featuring 3D lesion annotations in four key organs of the chest and abdomen: the liver, colon, pancreas, and lung. For our evaluation process, we chose to work with the volumes from the training set, as the testing set volumes did not include labels. The distribution of the organs with labels in the selected volumes was as follows: liver (131 volumes), lung (64 volumes), colon (126 volumes), and a total of 281 volumes containing lesions. Figure 1(a) shows the main steps for the dataset construction using a liver use case as an example.

For segmentation of relevant structures, the comprehensive multi-organ semantic segmentation model, TotalSegmentator^12^, is leveraged, which is based on U-Net-like architectures^36,38^.

### Data processing

In the following we outline each processing step to construct the benchmark. The 3D-MIR dataset is intentionally designed to facilitate the evaluation of both broad (such as distinguishing between organs with and without lesions) and detailed queries (like the number and size of lesions in each organ). It is adaptable for integration with existing public datasets that already include lesion labels. Our intention is to make this dataset publicly available in conjunction with the camera-ready version of this paper.

An expert radiologist validated a random subset of the 3D-MIR dataset, based on the volumes only (without additional test results). We developed an interface (cf. Fig. 2) to allow visualizing the 3D volumes and manually validating the tumor flag and group annotations.

#### Step 1: Organ indexing

We begin by applying TotalSegmentator to each CT volume, which generates 104 cropped organ-CT volumes and corresponding labels for each subject^12^. These cropped volumes are defined by 3D bounding boxes, each encompassing the organ of interest. This initial segmentation achieves two key objectives: it localizes lesions within a comprehensive anatomical ontology and streamlines the indexing process for organ-specific queries, enhancing the efficiency and precision of data retrieval.

#### Step 2: Lesion indexing

In this step, we analyze the 3D label volume associated with each CT scan to identify lesions. Given that 3D lesions are represented in a binary volume format, distinguishing lesion structures from the surrounding anatomy, we employ single-component connectivity analysis. This method effectively segregates individual lesions, allowing us to assign unique identifiers to each lesion and the corresponding subject. Subsequently, we perform quantification of each lesion’s spatial overlap with the 104 organs segmented by TotalSegmentator. This quantification is crucial as it enables us to accurately map each lesion to its respective organ and lesion distribution within the organ. Such mapping is instrumental in creating a robust and precise dataset, facilitating targeted organ-specific analyses and queries.

#### Step 3: Lesion morphology extraction

In this step, we focus on extracting morphological characteristics of each lesion identified in the previous steps by analyzing the 3D and 2D geometry of lesions localized in each organ. Furthermore, we extract morphological metrics corresponding to:

**a. Subject-organ.** Subjects were grouped based on the morphological characteristics of their lesions, following the guidelines set by the American Joint Committee on Cancer’s Tumor, Node, Metastasis (TNM) classification system^39^. This grouping adheres to the TNM system’s recommendations for classifying cancer stages and provides a standardized framework for correlating lesion morphology with cancer stage. Our focus here is primarily on lesion morphology and distribution, rather than on cancer staging. Specifically, we consider the number, length, and volume of the lesions to define three groups:

**Lesion group 1:** subjects with a single lesion smaller than 2 cm.
**Lesion group 2:** subjects with either a single lesion larger than 2 cm or multiple lesions, with none exceeding 5 cm.
**Lesion group 3:** subjects with one or more lesions, each larger than 5 cm.

**b. Single 3D lesion.** For each distinct lesion, we calculate comprehensive 3D morphological descriptors. These descriptors are crucial in understanding the lesion’s characteristics, including: 1) Lesion topology, particularly focusing on symmetrical shapes, 2) the volume of the lesion in actual physical space, the size of the lesion, represented through the dimensions of a fitted 3D ellipsoid. For the extraction of these metrics, we have employed the ITK (Insight Segmentation and Registration Toolkit)^40^.

**c. Single 2D slices.** To complement 3D morphological analysis, we quantify 2D slices associated with lesions. In each selected slice, we measure the total area occupied by lesions, count the number of distinct lesions, and analyze their morphology, focusing on characteristics such as circularity, allowing slice-based retrieval.

## Retrieval methods

**Fig. 3:**
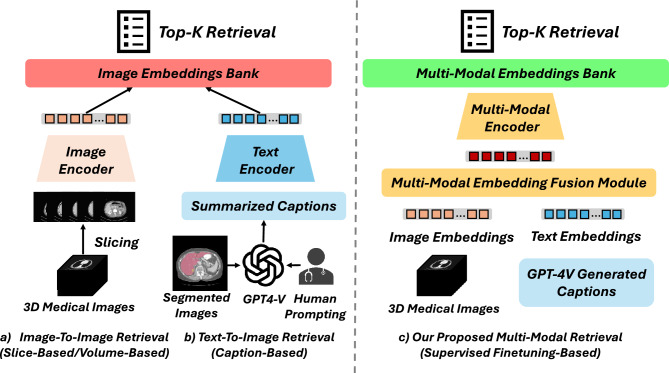
Overview of the proposed 3D image search approaches: Image-to-Image (slice-based or volume-based retrieval), Text-to-Image (caption-based), and multi-modal search methods (caption-volume-based or our proposed supervised fine-tuning-based approach, M2ST).

We investigate a diverse assortment of search approaches and compare the costs and benefits of each. Specifically, we first examine two query formats: sets of 2D slices versus single 3D volume. Second, we compare the performance of various approaches to evaluate the relevance and difficulty of the benchmark. Finally, we describe and evaluate a new multi-modal ensemble method.

### Preliminaries

In our study we rely on the BiomedCLIP vision-language model^28^ to generate embeddings for both the 2D images and their captions. BiomedCLIP is pretrained on PMC-15M, a dataset of 15 million biomedical image-text pairs from over three million articles. The input images consist of 2D slices extracted from CT volumes, with voxel values represented in Hounsfield Units (HU). For processing, we normalize these 3D volumes from the original range of [-1000, 1000] HU to a scale of 8-bit values ranging from [0, 255]. Subsequently, from these normalized values, we generate a monochromatic image.

### Image-to-image search methods

#### Slice-based retrieval

This search method relies on the 2D slices of the 3D volumes. Each 2D slice is encoded using embeddings from the model, as shown in Fig. 3. All 2D slices of all volumes are indexed in a vector database using the Faiss library^18^.

In the search step, for a given 3D volume input *V*, the slice-based retrieval consists of using all the 2D slices of *V* as queries (noted $Q_V$) that we use to search the vector database to retrieve the top 20 similar slices for each slice query in $Q_V$ based on the Euclidean distance.

The retrieved 2D slices are then aggregated to their parent 3D volumes to return top-k similar images. We compared three different slice results aggregation methods based on frequency, maximum similarity score, and sum of similarity scores of the retrieved slices $R(Q_V)$ for each 3D image that has slices in the results.

Noting the retrieved slices for a given query $Q_V$ as $R(Q_V)$, and the set of retrieved slices belonging to the same volume $V_i$ as $RS(V_i)$, the three aggregation methods rely on the following parent volume scoring methods:

1 $$\begin{aligned} \text {Freq}(V)= & \frac{|RS(V_i)|}{|R(Q_V)|} \end{aligned}$$

2 $$\begin{aligned} \text {MaxScore}(V)= & \max _{S_i \in RS(V_i)} \big (\text {SimScore}(S_i)\big ) \end{aligned}$$

3 $$\begin{aligned} \text {ScoreSum}(V)= & \sum _{S_i \in RS(V_i)} \big (\text {SimScore}(S_i)\big ) \end{aligned}$$

In each aggregation method, the parent volumes are scored then ranked according to the respective formulas in Equations 1, 2, and 3. The SimScore is based on the Euclidean distance between the embeddings of the slice query and the embeddings of a retrieved slice.

#### Volume-based retrieval

The volume-based retrieval relies on aggregating the embeddings of the 2D slices to generate one representative embedding vector for the whole volume. We compared four different embedding aggregation methods: median, max pooling, average pooling, and standard deviation. Each aggregation method *Mi* is used to generate a separate vector database/index (3D index). In the search step, we generate the embeddings of a query volume *V* according to *Mi* and search for the top-k similar volumes/vectors in the corresponding 3D index.

### Text-to-image search methods

#### Caption generation

To create natural language queries that mimic the text that radiologists would use to query the database, we built three methods to generate captions from 3D volumes:

- **CapGen I:** Our first caption generation method relies on a SimpleITK-based solution to extract the morphological/appearance-related features from the volumes. GPT-4, OpenAI’s latest GPT foundation model^37^, is then used to generate natural language captions based on the extracted image features: organ, number of lesions, and length of the largest lesion. The temperature parameter controls the randomness/creativity of the generated text. We selected a low temperature (temperature=0) to generate more focused/concise descriptions and to reduce hallucinations.
- **CapGen II:** The second method consists of generating captions to 2D slices using an encoder-decoder framework, based on the Convolutional Vision Transformer as the encoder and DistilGPT2 as the decoder, and fine-tuned on the ROCOv2 dataset of 80k radiology images and their captions^41^. The 2D slice-based captions are then summarized using GPT-4 to generate a caption for the 3D volume.
- **CapGen III:** The third caption generation method uses OpenAI’s latest model, GPT-4o, to generate captions from the 2D slices of the volume and then summarize the 2D captions to generate a 3D caption to the volume. To reduce execution time/cost and noise, we extract/use only one slice every three slices.

Table 1 presents examples of automatically generated captions from 3D volumes.

**Table 1:** Examples of generated captions using the CapGen I/II/III methods.

Volume	CapGen I	CapGen II	CapGen III
liver_29	This 3D volume depicts a liver with a single tumor measuring 14.69 centimeters.	The 3D volume of the CT scan reveals calcifications in several regions including the left temporal bone, left optic nerve, left renal vein, left maxillary sinus, left ventricular cavity and septum, and left pulmonary artery. Additionally, a mass is visible in the left ventricular cavity.	The 3D CT scan images encompass various anatomical regions, including the abdomen, pelvis, chest, neck, and brain. Notable abnormalities include bilateral subpleural nodules in the lungs, potential mucosal thickening in the sinuses, bilateral hypodense lesions on the liver suggestive of possible hepatic metastases, and an abnormal lesion on the right lung. Further clinical correlation and evaluation are recommended for these findings.
lung_042	This lung 3D volume reveals a single tumor measuring 6.75 centimeters, indicating a case of lung cancer.	The 3D volume of the CT scan reveals a recurring calcification in the left renal vein, a mass in the left renal vein, and calcifications in the left temporal bone. There is also a noted calcification in the left pulmonary artery.	The 3D CT scan encompasses the chest, abdomen, and pelvis, displaying various anatomical structures including the lungs, heart, liver, spleen, kidneys, and vertebrae. There is a noted bilateral lung pathology in the chest images, suggesting abnormalities that require further evaluation.

#### Text-to-image retrieval

**Caption-based method.** We used CapGen I captions to query the 2D slice index described in Section Slice-based retrieval through the BiomedCLIP vision-language model. The vision-language model was trained to maximize the similarity between the caption embeddings and the image embeddings using contrastive loss^28^. The results are then aggregated from the 2D slice level to the parent 3D images, based on the frequency (Equation 1) of the retrieved slices to return top-k similar volumes.

**Ensemble method.** The ensemble method consists of combining search results from the caption-based method and the frequency-based slice search. The combination is based on interleaving the volumes returned by each method according to their ranks.

### Multi-modal search methods (M2ST)

**Fig. 4:**
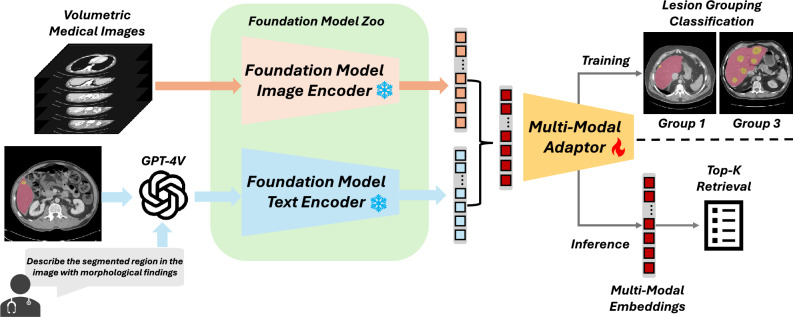
Overview of supervised finetuning with multi-modal embeddings for 3D image retrieval. We first select modality-specific foundation models from the foundation models in Azure AI Model Catalog. We then generate embeddings using both an image encoder and a text encoder from a foundation model. These embeddings are fused via element-wise multiplication, and the combined output is used to train a lightweight adaptor model, such as an MLP. Lesion group categories serve as supervised labels to enhance the model’s semantic understanding of tumor morphology. The output feature embeddings from the adaptor are then utilized for direct ranking.

In addition to traditional image-to-image and text-to-image retrieval approaches, we introduce a multi-modal retrieval framework, depicted in Fig. 4, that unifies image and text data into a shared embedding space for efficient and accurate multi-modal search. The framework begins by encoding 3D medical images and their corresponding textual descriptions using a combination of foundation models: image encoders like BiomedCLIP and MedImageInsight, along with the BiomedCLIP text encoder. Vision embeddings are then processed through a small stack of MLP layers to downsample and align their dimensions with the text embeddings, facilitating seamless multi-modal fusion. Inspired by Zala *et al.*^42^, we employ element-wise multiplication to merge the transformed vision embeddings with the text embeddings, incorporating an attentional mechanism that emphasizes regions of interest referenced in the text. The fused embeddings are subsequently fine-tuned in a multi-modal supervised training (M2ST) process, where adapters are optimized using lesion groupings from 3D-MIR as ground-truth labels. We hypothesize that these enhanced embeddings effectively capture semantic information necessary for retrieving images with similar lesion groupings. During inference, we compute embeddings for both image and text inputs, using cosine similarity to retrieve top-k similar images from the multi-modal embedding bank.

Additionally, we hypothesize that the semantic content provided by text descriptions influences adaptor training convergence, with vision embeddings being sensitive to variations in text embeddings. To examine the impact of text semantics variability, we conduct M2ST using different versions of CapGen, evaluating the model’s performance on lesion group classification accuracy and retrieval precision. These experiments aim to elucidate the sensitivity induced by text descriptions and establish benchmarks for the effectiveness of various semantic representations.

### Indexing and evaluation

In all our experiments, we constructed the 2D/3D index using the training slices and volumes from each organ-specific dataset. To make each collection more heterogeneous, the training and test sets were augmented with volumes randomly selected from the other datasets. For example, the Liver training set was augmented using volumes from the Colon, Pancreas, and Lung datasets with lesions less than 2 cm. This cross-organ augmentation integrates ‘specific-healthy’ organs into the data, helping to evaluate the model’s ability to distinguish subtle lesion features in a more diverse and realistic retrieval setting. Details of these training and test sets are outlined in Table 2.

**Table 2:** Training-test splits for each dataset/organ: The training volumes/slices are used to create the 3D/2D index and the test volumes are used as test queries.

Dataset (Organ)	Training Set:	Test Set:
	#volumes / #slices	#volumes
Liver	157 / 58,982	19
Colon	209 / 32,732	24
Pancreas	269 / 31,785	32
Lung	94 / 23,587	47

**Fig. 5:**
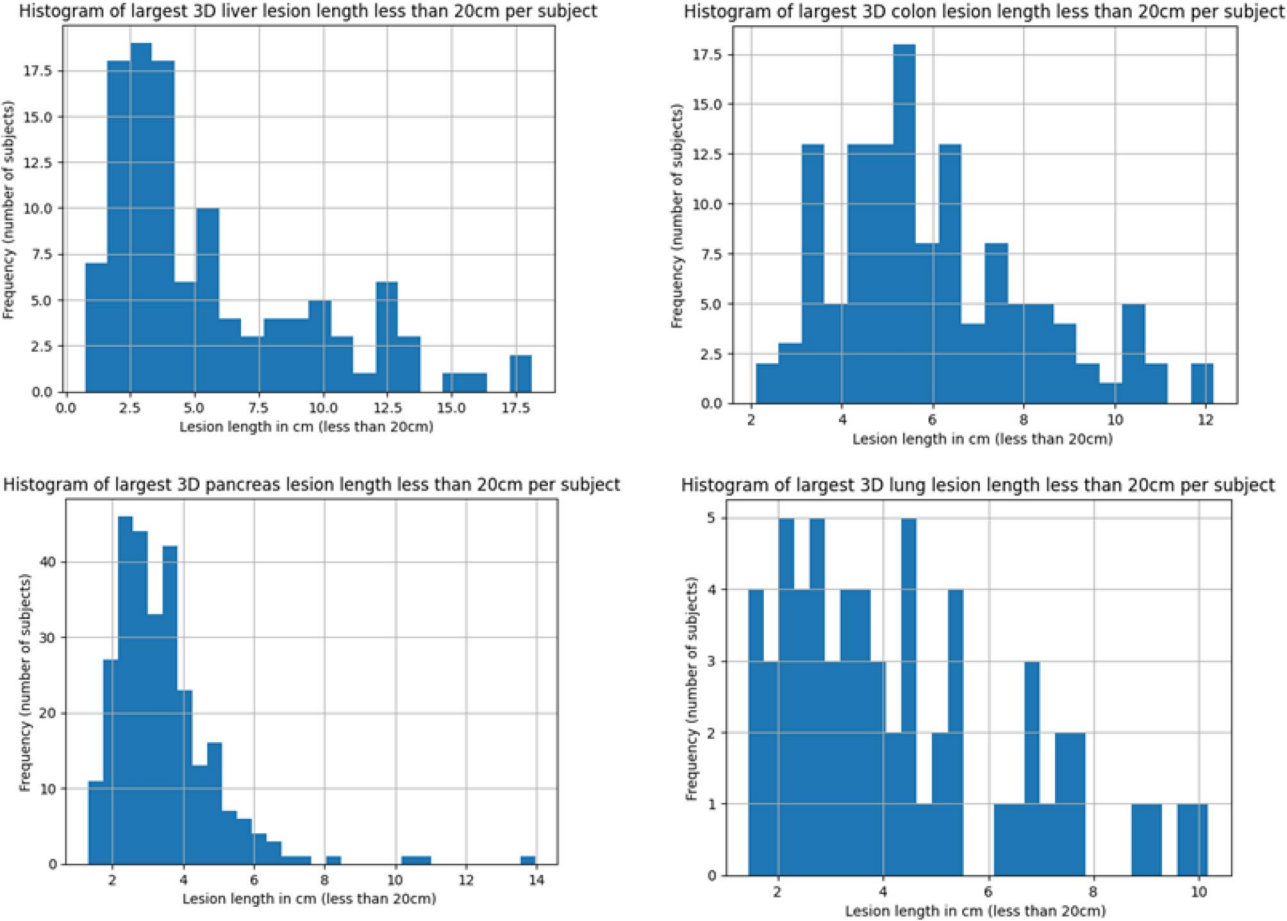
Lesion size distribution across organs: Liver, Colon, Pancreas, and Lung.

Additionally, Fig. 5 illustrates the size distribution of the largest lesions, measured in centimeters, across different organs, highlighting the variability in lesion size depending on the organ.

We evaluate each search method by comparing the lesion flag and lesion group of the query volume and the top-k retrieved volumes. We then compute Precision@k (P@k) and Average Precision (AP), defined as:

4 $$\begin{aligned} AP = \sum _n (R_n - R_{n-1}) P_n \end{aligned}$$

with $R_n$ and $P_n$ are the Precision and Recall at the nth threshold.

**Fig. 6:**
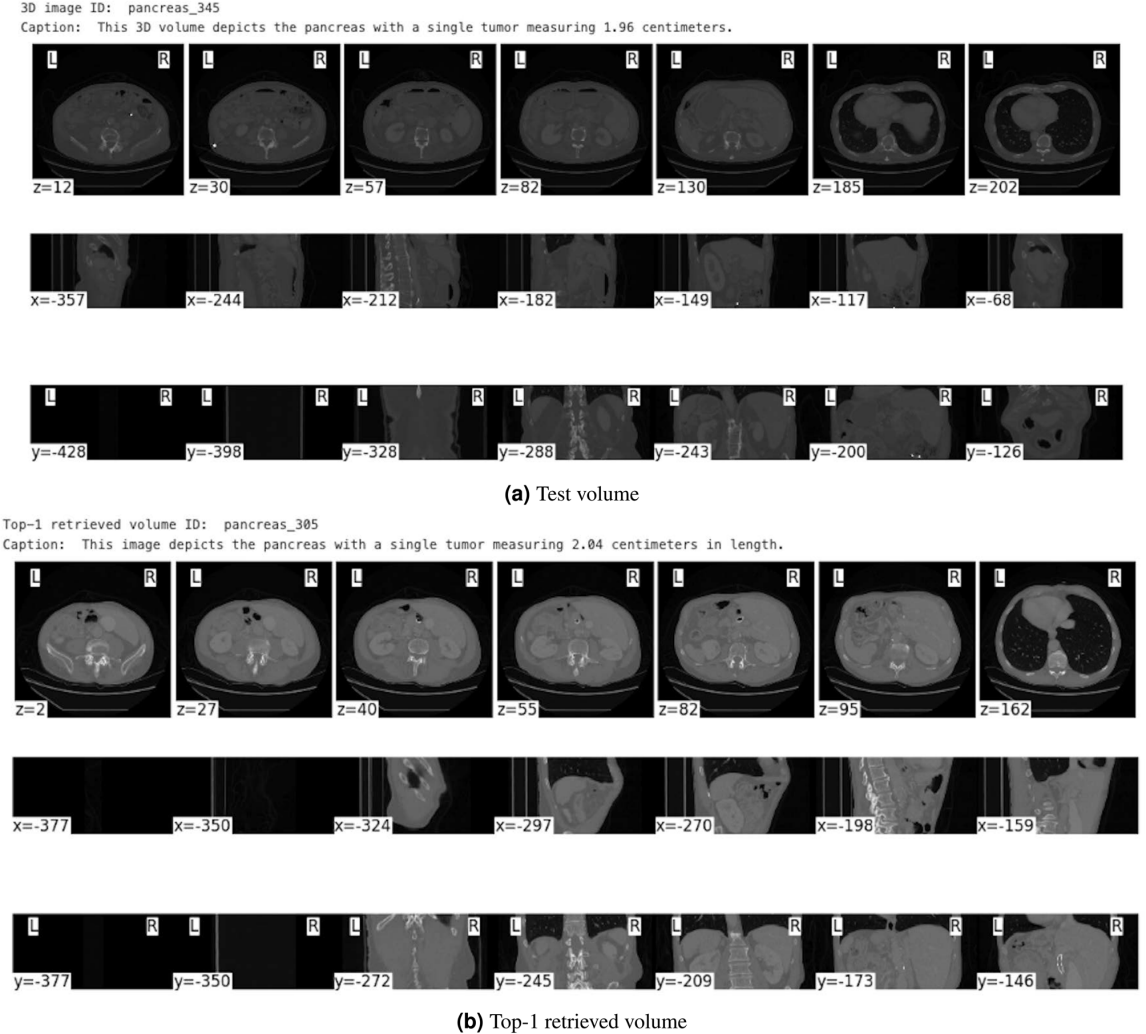
Example from the Pancreas dataset.

Figure 6 presents a test volume/query from the Pancreas dataset and the top-1 retrieved volume by the search methods.

## Experiments

### Our results

The overall results are presented in Table 3 and the specific organ-wise results are listed in Table 4, Table 5, Table 6, and Table 7 for the Liver, Colon, Pancreas, and Lung, respectively.

**Table 3:** Average Results across 3D-MIR Datasets (Liver, Colon, Pancreas, & Lung).

Average across all Datasets		Lesion Flag				Lesion Group			
Methods		P@3	P@5	P@10	AP	P@3	P@5	P@10	AP
			**Without Fine-tuning**						
Image-to-Image:	Frequency	86.5	86.2	85.1	90.5	61.5	58.1	54.4	69.6
Slice-based	Score/Max	84.8	84.6	84.5	89.0	52.5	50.0	50.0	64.4
	Score/Sum	86.5	86.0	85.1	90.4	61.7	57.9	54.6	69.5
Image-to-Image:	Median	90.2	89.2	87.7	91.4	53.3	52.6	52.7	64.6
Volume-based	MaxPooling	39.5	40.5	48.5	52.7	27.9	27.7	30.7	39.6
	AvgPooling	88.3	87.0	86.5	90.4	59.1	54.8	53.0	67.3
	StandardDev	53.7	59.3	58.0	61.4	32.0	31.6	29.2	39.8
Text-to-Image:	Caption	58.1	55.5	55.9	66.2	38.0	37.5	37.6	47.9
	Ensemble	76.8	74.9	71.9	82.6	55.1	53.9	51.9	65.2
			**With Fine-tuning**						
Multimodal:	CapGen I	**98.7**	**98.7**	**98.7**	**98.7**	**71.8**	**71.2**	**71.6**	**75.1**
M2ST	CapGen II	94.7	94.9	94.9	94.8	64.7	65.7	65.1	66.8
	CapGen III	95.7	95.6	95.5	95.7	66.5	66.5	65.8	68.7

**Table 4:** Precision@k and average precision on the liver dataset.

Liver		Lesion Flag				Lesion Group			
Methods		P@3	P@5	P@10	AP	P@3	P@5	P@10	AP
			**Without Fine-tuning**						
Image-to-Image:	Frequency	82.5	81.0	78.2	88.0	57.9	51.6	49.2	64.4
Slice-based	Score/Max	71.9	74.7	76.6	79.9	43.9	41.0	42.0	55.1
	Score/Sum	82.5	81.0	78.2	87.5	57.9	51.6	49.2	64.0
Image-to-Image:	Median	78.9	77.9	76.9	81.6	40.3	43.2	44.6	50.2
Volume-based	MaxPooling	68.4	61.0	65.4	69.7	22.8	25.3	29.1	40.6
	AvgPooling	73.7	74.7	74.7	80.2	47.4	48.4	45.5	55.6
	StandardDev	68.4	68.4	68.4	68.4	40.3	34.7	32.3	41.2
Text-to-Image:	Caption	70.2	61.0	61.0	77.9	36.8	34.7	34.7	53.6
	Ensemble	84.2	81.0	72.4	83.7	50.9	50.5	43.8	59.3
			**With Fine-tuning**						
Multimodal:	CapGen I	**94.7**	**94.7**	**94.7**	**94.7**	**70.2**	**70.5**	**72.4**	**73.7**
M2ST	CapGen II	84.2	84.2	84.2	84.2	50.9	51.6	51.1	53.9
	CapGen III	84.2	84.2	84.2	84.2	61.4	58.9	59.4	61.1

**Table 5:** Precision@k and average precision on the colon dataset.

Colon		Lesion Flag				Lesion Group			
Methods		P@3	P@5	P@10	AP	P@3	P@5	P@10	AP
			**Without Fine-tuning**						
Image-to-Image:	Frequency	70.8	74.2	73.8	79.5	55.6	54.2	51.5	62.7
Slice-based	Score/Max	77.8	76.7	75.5	86.0	50.0	46.7	45.5	61.5
	Score/Sum	70.8	73.3	73.7	79.3	55.6	54.2	51.9	62.4
Image-to-Image:	Median	84.7	83.3	78.8	87.1	51.4	51.7	47.9	63.3
Volume-based	MaxPooling	41.7	46.7	48.6	58.3	41.7	40.0	34.7	49.6
	AvgPooling	84.7	78.3	77.6	85.9	56.9	50.0	48.9	67.1
	StandardDev	58.3	60.8	57.0	63.7	23.6	25.0	25.0	35.7
Text-to-Image:	Caption	45.8	45.8	45.8	60.5	36.1	36.1	36.1	45.3
	Ensemble	65.3	61.7	59.4	69.8	55.6	49.2	46.3	56.2
			**With Fine-tuning**						
Multimodal:	CapGen I	**100**	**100**	**100**	**100**	**72.2**	**71.7**	**72.4**	**73.2**
M2ST	CapGen II	95.8	95.8	95.8	95.8	61.1	62.5	61.7	63.8
	CapGen III	98.6	98.3	97.9	98.8	63.9	65.0	64.6	67.8

**Table 6:** Precision@k and average precision on the pancreas dataset.

Pancreas		Lesion Flag				Lesion Group			
Methods		P@3	P@5	P@10	AP	P@3	P@5	P@10	AP
			**Without Fine-tuning**						
Image-to-Image:	Frequency	96.9	95.0	95.8	98.8	55.2	55.6	54.5	65.8
Slice-based	Score/Max	95.8	95.6	94.5	95.7	49.0	51.9	52.7	63.2
	Score/Sum	96.9	95.0	95.8	98.9	56.2	55.0	54.7	66.1
Image-to-Image:	Median	97.9	97.5	96.9	97.9	47.9	50.6	56.6	61.9
Volume-based	MaxPooling	12.5	12.5	32.7	30.1	12.5	12.5	25.6	24.5
	AvgPooling	96.9	96.9	96.6	97.1	58.3	55.6	57.6	63.2
	StandardDev	54.2	61.2	64.7	64.2	30.2	26.9	25.2	39.7
Text-to-Image:	Caption	82.3	81.2	82.6	83.3	44.8	45.0	45.6	57.1
	Ensemble	90.6	90.0	88.6	92.7	46.9	48.7	50.4	58.7
			**With Fine-tuning**						
Multimodal:	CapGen I	**100**	**100**	**100**	**100**	62.5	61.9	63.6	68.9
M2ST	CapGen II	99.0	99.4	99.4	99.2	**72.9**	**74.4**	**72.9**	**74.1**
	CapGen III	**100**	**100**	**100**	**100**	68.7	70.0	67.2	71.1

**Table 7:** Precision@k and average precision on the lung dataset.

Lung		Lesion Flag				Lesion Group			
Methods		P@3	P@5	P@10	AP	P@3	P@5	P@10	AP
			**Without Fine-tuning**						
Image-to-Image:	Frequency	95.7	94.5	92.7	95.9	77.3	71.1	62.5	85.7
Slice-based	Score/Max	93.6	91.5	91.3	94.5	67.4	60.4	59.8	77.9
	Score/Sum	95.7	94.5	92.7	95.9	77.3	71.1	62.5	85.5
Image-to-Image:	Median	99.3	98.3	98.3	99.1	73.8	65.1	61.8	83.1
Volume-based	MaxPooling	35.5	41.7	47.5	52.9	34.7	33.2	33.6	43.6
	AvgPooling	97.9	97.9	96.9	98.5	73.8	65.1	60.1	83.3
	StandardDev	34.0	46.8	42.0	49.4	34.0	40.0	34.4	42.7
Text-to-Image:	Caption	34.0	34.0	34.0	43.0	34.0	34.0	34.0	35.6
	Ensemble	67.0	67.0	67.0	84.1	67.0	67.0	67.0	**86.6**
			**With Fine-tuning**						
Multimodal:	CapGen I	**100**	**100**	**100**	**100**	**82.3**	**80.6**	**78.1**	84.7
M2ST	CapGen II	**100**	**100**	**100**	**100**	74.0	74.4	74.7	75.3
	CapGen III	**100**	**100**	**100**	**100**	71.9	71.9	71.9	74.9

**Table 8:** 3D Image retrieval performance using sifferent foundation models.

Model	Lesion Flag				Lesion Group			
	P@3	P@5	P@10	AP	P@3	P@5	P@10	AP
	Liver							
BiomedCLIP	79.0	77.9	76.9	81.6	40.4	43.2	44.6	50.2
Med-Flamingo	42.1	45.3	47.7	50.6	35.1	35.8	36.8	41.4
BiomedGPT	**82.5**	**81.0**	79.6	**84.0**	**45.6**	**49.5**	**46.1**	**55.4**
Curia	77.2	75.8	73.9	80.6	**47.4**	43.2	42.7	53.3
MedImageInsight	80.7	77.9	**81.7**	82.3	43.9	42.1	44.5	54.6
	Colon							
BiomedCLIP	84.7	83.3	78.8	87.1	51.4	51.7	47.9	63.3
Med-Flamingo	54.2	60.00	53.9	69.6	36.1	42.5	36.1	54.9
BiomedGPT	59.7	52.5	48.3	80.5	50.0	46.7	44.4	59.6
Curia	91.7	88.3	83.8	97.2	55.6	52.5	49.1	67.7
MedImageInsight	**100**	**99.2**	**97.3**	**99.7**	**65.3**	**62.5**	**60.6**	**69.4**
	Pancreas							
BiomedCLIP	97.9	97.5	96.9	97.9	47.9	50.6	56.6	61.9
Med-Flamingo	94.8	93.1	92.3	94.9	**64.6**	**58.1**	53.6	65.6
BiomedGPT	**100**	**100**	**99.4**	**100**	44.8	51.9	**57.8**	61.1
Curia	97.9	97.5	97.2	97.9	57.3	55.6	54.8	64.4
MedImageInsight	**100**	**100**	**99.4**	99.9	60.4	**58.1**	57.6	**67.3**
	Lung							
BiomedCLIP	99.3	98.3	**98.3**	99.1	**73.7**	65.1	61.8	**83.1**
Med-Flamingo	96.9	96.9	95.5	97.3	70.8	69.4	66.8	75.6
BiomedGPT	**100**	**100**	94.1	**100**	72.0	72.9	66.2	76.2
Curia	97.9	96.2	93.4	97.7	69.8	69.4	66.5	75.1
MedImageInsight	**100**	**100**	96.9	99.7	69.8	**73.8**	**68.6**	76.1
	Average across all organs							
BiomedCLIP	90.2	89.3	87.7	91.4	53.4	52.6	52.7	64.6
Med-Flamingo	72.0	73.8	72.3	78.1	51.7	51.4	48.3	59.3
BiomedGPT	85.5	83.4	80.4	91.1	53.1	55.2	53.6	63.1
Curia	91.2	89.4	87.1	93.3	57.5	55.2	53.3	65.1
MedImageInsight	**95.2**	**94.3**	**93.8**	**95.4**	**59.8**	**59.1**	**57.8**	**66.9**

**Table 9:** Results of the expert-based clinical relevance evaluation of retrieved images.

*k*	P@k ($=3$)	P@k ($\ge 2$)	nDCG@k
1	0.600	1.000	1.000
2	0.625	1.000	0.979
3	0.650	1.000	0.962

**Table 10:** M2ST-CapGenI Results using BiomedCLIP vs. MedImageInsight.

M2ST-CapGenI Method	Lesion Flag				Lesion Group			
	P@3	P@5	P@10	AP	P@3	P@5	P@10	AP
	Liver							
M2ST-BiomedCLIP	**94.7**	**94.7**	**94.7**	**94.7**	70.2	70.5	**72.4**	73.7
M2ST-MedImageInsight	84.2	84.2	85.2	85.1	**77.2**	**71.6**	69.7	**77.6**
	Colon							
M2ST-BiomedCLIP	**100**	**100**	**100**	**100**	72.2	71.7	72.4	73.2
M2ST-MedImageInsight	**100**	**100**	**100**	**100**	**76.4**	**75.8**	**77.0**	**79.2**
	Pancreas							
M2ST-BiomedCLIP	**100**	**100**	**100**	**100**	62.5	61.9	63.6	68.9
M2ST-MedImageInsight	**100**	**100**	99.7	**100**	**74.0**	**73.1**	**72.6**	**78.0**
	Lung							
M2ST-BiomedCLIP	**100**	**100**	**100**	**100**	82.3	80.6	78.1	84.7
M2ST-MedImageInsight	99.0	98.1	96.4	99.6	**93.7**	**93.7**	**93.7**	**93.7**
	Average across all organs							
M2ST-BiomedCLIP	**98.7**	**98.7**	**98.7**	**98.7**	71.8	71.2	71.6	75.1
M2ST-MedImageInsight	95.8	95.6	95.3	96.2	**80.3**	**78.6**	**78.2**	**82.2**

We compare three main approaches *without fine-tuning*: Slice-based, Volume-based, and Text-to-Image methods. The **lesion flag** is best recognized by the volume-based search method with median pooling (used to define the volume embeddings) in three out of four datasets. This demonstrates the benefits of using the 3D context to accurately detect lesions. Given that ground truth data was generated using a classification model, the substantially better performance of the volume-based search method was likely due to reduced signal noise over the individual 2D slice embeddings. The **lesion group** is most effectively identified by either the slice-based search method or the multi-modal search method in three out of the four datasets. This is likely because the lesion group frequently correlates to its size relative to the organ (lesion size distribution is shown in Fig. 5). A cumulative perspective (such as the sum) of the data in the individual slices appears to better represent the lesion’s significance within the organs. In contrast to a volume-based representation which aggregates embeddings from all the slices into a single global semantic representation, potentially losing the detailed cumulative information about the lesion’s relative size.

The text-to-image ensemble method, combining the results of the caption-based and the slice-based methods, achieved better precision@3 of 84.21% for lesion flag matching on the Liver dataset and better precision@10 of 67.02% and average precision of 86.61% for lesion group matching on the Lung dataset. However, overall, this ensemble with results fusion underperformed compared to the slice-based and volume-based methods. This is likely due to the relatively low similarity between the caption embeddings and the image embeddings. The new multi-modal supervised training (M2ST) approach achieved the best precision scores (P@3, P@5, P@10, and Average Precision) for both lesion flag and lesion group matching in all datasets/organs (Liver, Colon, Pancreas, and Lung). 

We also study the volume-wise search method when using different foundation models. In addition to BiomedCLIP (used in all previous experiments), we selected Med-Flamingo^43^, and the new vision-language models BiomedGPT^44^, Curia^47^ and MedImageInsight^45^.

Table 8 presents the results of the volume-based search method (median) using these models. BiomedCLIP-based retrieval outperforms solutions based on the other models in lesion presence matching (P@10) and lesion group matching (P@3 and Average Precision) in one out of the four datasets. When considering the macro-average across all datasets, MedImageInsight-based retrieval outperforms substantially all other models in all evaluation metrics (Lesion Presence/Staging Precision@k and Average Precision). Table 10 presents the results of the M2ST-CapGenI method using BiomedCLIP vs. MedImageInsight embeddings. The M2ST-CapGen I model has the best precision scores in lesion flag matching when using BiomedCLIP embeddings (98.69% vs. 96.18% Average Precision) and achieves higher scores in lesion group matching using MedImageInsight embeddings (82.16% vs. 75.13% Average Precision).

**Table 11:** HiREST: Ablation of text inputs (captions).

Model	Lesion Flag				Lesion Group			
	P@3	P@5	P@10	AP	P@3	P@5	P@10	AP
	M2ST - with Text Input (Caption I)							
Liver	**94.7**	**94.7**	**94.7**	**94.7**	**70.2**	**70.5**	**72.4**	**73.7**
Colon	**100**	**100**	**100**	**100**	**72.2**	71.7	72.4	73.2
Pancreas	**100**	**100**	**100**	**100**	**62.5**	**61.9**	**63.6**	**68.9**
Lung	**100**	**100**	**100**	**100**	**82.3**	**80.6**	**78.1**	**84.7**
	M2ST - without Text							
Liver	87.7	86.3	85.4	88.5	47.4	49.5	50.4	54.3
Colon	94.4	95.0	95.4	94.9	**72.2**	**73.3**	**73.3**	**74.5**
Pancreas	96.9	96.9	96.9	96.9	55.2	60.6	60.6	64.6
Lung	**100**	**100**	**100**	**100**	71.9	72.5	72.8	79.4

Table 11 presents the results of the ablation study for the multi-modal (M2ST) approach when using (or not) text inputs/captions. With caption inputs, the performance of the M2ST model increases based on lesion flag matching in all datasets as well as lesion group matching in three out of four datasets.

### 3D Embedding models

We compared the efficacy of retrieval using our 2D MedImageInsight method with 3D embedding models such as M3D ^46^. Under this setting, we evaluated both organ lesion detection and severity assessment, with a particular emphasis on pancreatic lesions. We used our 3D-MIR dataset focusing on the pancreas, with the same training and testing splits as described previously (the code is available at: https://github.com/microsoft/healthcareai-examples/blob/main/azureml/advanced_demos/image_search/3d_image_search.ipynb).

For image pre-processing, we followed the recommended normalization pipeline from M3D by scaling the image volumes to the $[0,1]$ range using min–max normalization. For M3D, all images were resized to $256 \times 256$, and stacks of 32 slices were extracted to form tensors of shape $(n, 32, 256, 256)$, where $n$ is the number of stacks per 3D volume. Each stack $n$ was passed through the M3D image encoder, and the resulting embeddings were then averaged across all stacks to produce a single 3D embedding per volume.

For MedImageInsight, we applied the encoder to each image and then averaged the embeddings across the images as described earlier. This ensured that both models were evaluated under comparable conditions.

While 3D foundation models are designed to capture spatial context across slices, they are fundamentally constrained in their ability to encode an entire 3D volume at once due to memory and input size limitations. As a result, most current 3D methods rely on aggregation (e.g., averaging) or concatenation of fixed-size stacks, rather than holistic volumetric analysis. One key challenge is the selection of slice stacks, which lacks standardization and can have a substantial impact on model performance. This introduces variability and makes reproducibility harder in clinical deployments.

In contrast, 2D methods like MedImageInsight are not only easier to implement and operationalize (leveraging widely adopted 2D CNN or vision transformer backbones), but also avoid arbitrary stack selection and can process the entire image dataset slice-by-slice, preserving resolution and interpretability. These features make 2D methods highly effective, particularly when coupled with intelligent embedding strategies and averaging techniques.

Table 12 summarizes the overall comparison of lesion detection by group. MedImageInsight achieves consistently higher overall precision at all top-$k$ values (Top-1: 0.5 vs. 0.4; Top-3: 0.5 vs. 0.3; Top-5: 0.5 vs. 0.3) compared to M3D. In particular, MedImageInsight outperforms M3D for Healthy cases (1.0 vs. 0.0) and for Lesion Group 3 at Top-3 and Top-5 (0.3 vs. 0.2). By contrast, M3D achieves slightly higher precision on Lesion Group 1 and Lesion Group 2 at Top-$k$, reflecting its 3D context sensitivity.

**Table 12:** Precision comparison between M3D and MedImageInsight across lesion categories and Top-K values. Best results are in bold.

Category	M3D			MedImageInsight		
	P@1	P@3	P@5	P@1	P@3	P@5
Healthy	0.0	0.0	0.0	**1.0**	**1.0**	**1.0**
Lesion Group 1	**0.3**	**0.2**	**0.2**	0.0	0.0	0.1
Lesion Group 2	**0.9**	**0.8**	**0.8**	0.7	0.7	0.7
Lesion Group 3	0.3	0.2	0.2	**0.3**	**0.3**	**0.2**
**Overall Precision (Top-K)**	0.4	0.3	0.3	**0.5**	**0.5**	**0.5**

## Clinical relevance evaluation

To evaluate the clinical relevance of retrieved volumes, a board-certified radiologist annotated a subset of retrieval outputs. We selected five random test volumes and their top-3 retrieved volumes by the M2ST-CapGenI-BiomedCLIP method. We used a 1-3 Likert scale for clinical relevance:

- 1 = Irrelevant (different organ or mismatched tumor characteristics).
- 2 = Borderline (same organ with partial overlap, but notable differences).
- 3 = Highly Relevant (strong match in organ, type, morphology, enhancement, etc.).

We computed three standard information retrieval metrics adapted to this graded relevance scale:

- Precision@k ($=3$): fraction of top-*k* images rated Highly Relevant (strict measure of clinical utility).
- Precision@k ($\ge 2$): fraction of top-*k* images rated at least Borderline (lenient measure capturing partial usefulness).
- nDCG@k: normalized discounted cumulative gain with mapping $\{1,2,3\} \rightarrow \{0,1,2\}$, rewarding higher-ranked Highly Relevant images.

Table 9 reports the results of the manual evaluation. We observe that Precision@k ($\ge 2$) is consistently 1.0 for $k=1,2,3$, indicating that all retrieved cases are at least partially useful. Precision@k ($=3$) increases from 0.60 at $k=1$ to 0.65 at $k=3$, showing that the system retrieves more Highly Relevant cases as the candidate set grows. nDCG@k remains high (1.0, 0.979, 0.962), confirming that Highly Relevant cases are consistently ranked near the top.

Overall, this expert-based evaluation demonstrates that the system is able to retrieve clinically meaningful reference cases with strong top-ranked performance.

## Discussion

**Fig. 7:**
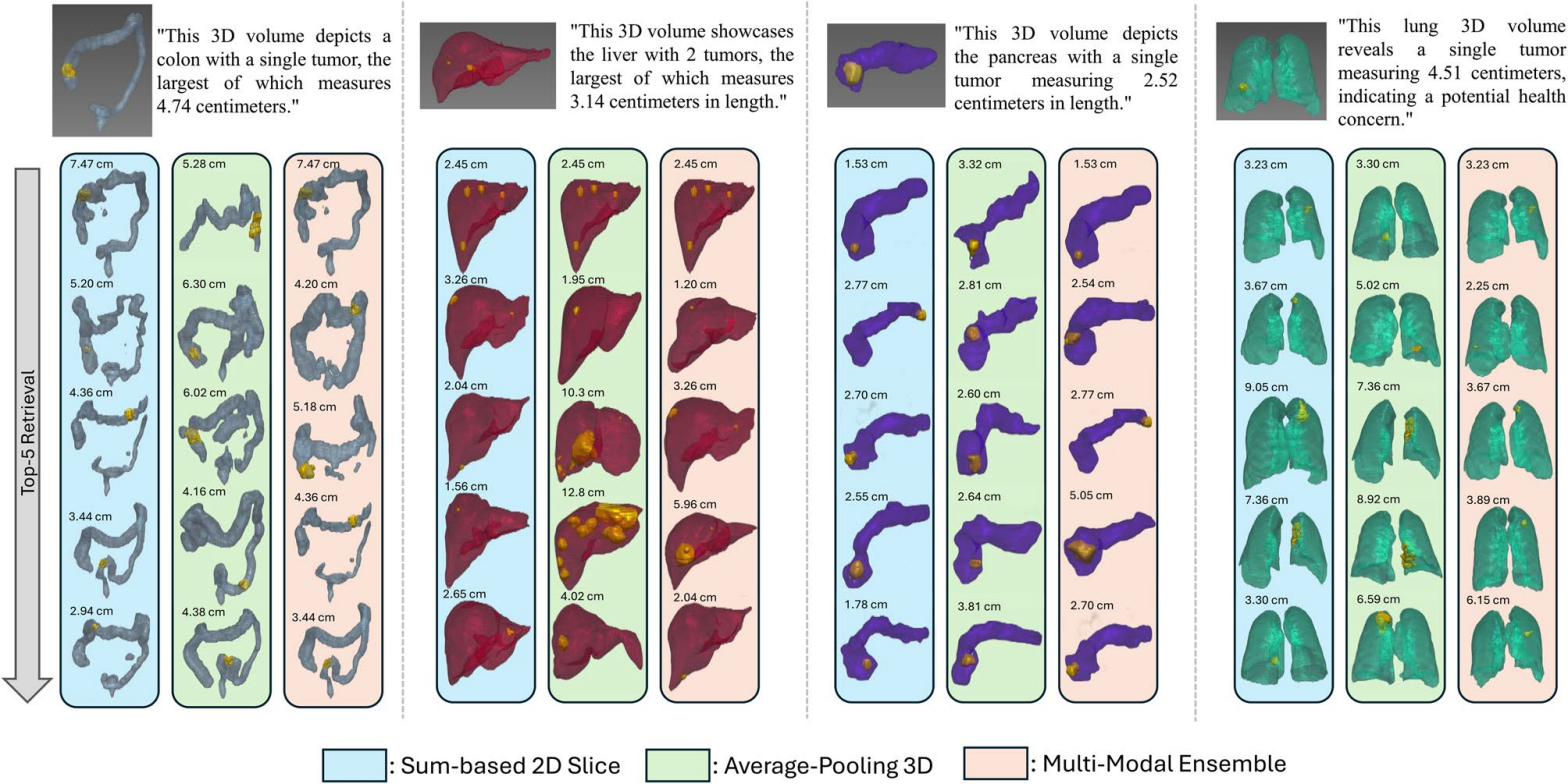
This figure showcases a qualitative visual representation of medical image retrieval outcomes, organized according to organ types (colon, liver, pancreas, lung) and corresponding text queries. The evaluation encompasses three methods: (i) the Sum-based 2D slice method, shown in blue to the left of each organ category, (ii) the Average Pooling-based 3D volume method, displayed in green at the center, and (iii) the text-to-image ensemble method, featured in pink on the right. The results display the top-5 ranked outputs for each method.

In our analysis, we not only measure quantitative aspects but also investigate how well different methods correlate organ retrieval with their lesions by creating a 3D surface visualization of lesion groups for each approach.

Figure 7 showcases a qualitative visual representation of medical image retrieval outcomes, organized according to organ types (colon, liver, pancreas, lung) and corresponding text queries. The evaluation encompasses three methods: (i) the Sum-based 2D slice method, shown in blue to the left of each organ category, (ii) the Average Pooling-based 3D volume method, displayed in green at the center, and (iii) the Text-to-Image ensemble method, featured in pink on the right. The results display the top-5 ranked outputs for each method.

Initially focusing on the liver, we find that both 2D slice-based and multi-modal methods show adeptness in identifying smaller lesions located near the liver’s periphery. In contrast, the 3D volumetric approach excels in retrieving images of larger lesions, despite showing a lower correlation between the query and the results.

When searching for colon lesions, the results are consistent across all methods, with each showing a strong correlation in terms of accurately determining the lesion group. However, we observe a variation in the accuracy of lesion location identification. The 2D slice-based method is particularly effective in locating lesions in the left section of the colon.

Regarding the pancreas, foundation model representations are notably successful in identifying lesion regions, especially at the lower end of the organ. Despite some limitations in classifying the group-size of pancreatic lesions (cf. Table 6), the 3D approach shows adaptability in handling complex lesion morphologies like U-shape configurations.

From our preliminary analysis of the 2D slice-based embedding, it appears that tasks involving broader categorization, such as lesion detection (lesion flag), are more effective than those requiring more detailed analysis, like lesion group classification.

Additionally, our findings indicate that no single method excels uniformly across all types of organs. This observation suggests that the variability between different organs significantly influences the effectiveness of encoding both lesion information and the appearance of the organs. In our initial experiments, we examined the use of 2D embedding techniques for complex 3D tasks. The performance issues we encountered might be linked to pre-analytical variables like image resolution and normalization, as well as to biological and anatomical differences, such as varying organ sizes and distinct organ characteristics. Despite applying 2D embeddings in scenarios beyond their typical domain, they proved to be useful, particularly in identifying specific morphological features and patterns in 3D data. This indicates that, although 2D embeddings may not completely capture the nuances of 3D tasks, they can still significantly enhance analysis, especially when integrated with other dimensional techniques.

## Conclusion

This paper introduces the first benchmark for 3D Medical Image Retrieval (3D-MIR), covering four types of anatomies imaged via computed tomography, and evaluates a range of search strategies leveraging popular state-of-the-art multi-modal foundation models. These strategies involve queries based on aggregated 2D slices, 3D volumes, or multi-modal embeddings. Our findings indicate that while current foundation models are adept at identifying coarse-grained semantic details, such as lesion presence, they struggle with fine-grained information, such as the grouping of lesions by size. Given that the existing foundation models are designed for 2D inputs, our research concentrates on investigating two approaches to combine 2D information into a 3D context for 3D image search: slice-based and volume-based. Our experimental outcomes hint that volume-based approaches may be more effective for broad categorizations, whereas slice-based methods could be a more effective choice for capturing fine-grained details. Finally, our results demonstrate the effectiveness of the new multi-modal supervised training approach for both lesion flag and lesion group matching in all organs. This benchmark is made publicly available to support continued progress in methods and foundation models for 3D image retrieval.

## Limitations

Our study has several limitations. First, we rely on the publicly available MSD dataset and the automated segmentation tool *TotalSegmentator* to generate organ and lesion annotations. While both are widely used and validated, the segmentation masks were not manually reviewed in all cases, which may introduce variability and impact retrieval accuracy. Also, the MSD dataset does not provide patient identifiers, which prevents explicit verification of patient-level separation; in this work we therefore ensured non-overlapping partitions at the volume level when constructing index and query sets.

Second, our text-to-image retrieval experiments rely on automatically generated captions. Although these captions are useful for controlled benchmarking, they may not fully reflect the nuance, variability, and ambiguity of real clinical text, which could affect the ability to apply findings or results from a study or experiment to broader clinical contexts.

Third, while we included comparisons with both 2D and 3D embedding models, each approach has inherent limitations. Current 3D foundation models remain constrained in their ability to process entire volumetric scans at once due to computational and architectural limitations. Most 3D methods rely on aggregation (e.g., averaging) or concatenation of fixed-size stacks, which do not capture the full volumetric context. Moreover, the slice selection strategy is not standardized and may significantly impact performance, introducing variability and limiting reproducibility.

In contrast, 2D methods do not take advantage of full volumetric information, which may limit their ability to capture complex spatial relationships. However, they benefit from simpler and more reproducible pipelines, avoid arbitrary stack selection, and can process the entire dataset slice-by-slice. As shown in our experiments, this enables 2D approaches to perform comparably to 3D models in several retrieval tasks, particularly when embeddings are aggregated intelligently across slices. Their relative simplicity and scalability also make them easier to operationalize and integrate into clinical workflows.

In this work, performance improvements are reported in absolute terms without statistical testing. While our results are consistent across multiple datasets and metrics, incorporating bootstrapped confidence intervals or significance testing would strengthen the reliability of these improvements. We plan to include such analyses in future work, particularly as the benchmark evolves and performance differences between methods become more subtle.

Despite these inherited limitations, our work is intended to provide a useful foundation for advancing methods in 3D medical image retrieval, rather than serving as a clinical validation dataset. To support the reliability of our results, we additionally conducted human validation of retrieval precision. While this represents an initial step, it paves the way for more comprehensive benchmarking efforts and the development of larger, clinically validated datasets that can drive progress in this important area.

## Data Availability

The code and datasets generated and analyzed during the current study are available in this repository: https://github.com/abachaa/3D-MIR.
